# Decoding the Link Between Talcum Powder and Ovarian Cancer: A Comprehensive Review

**DOI:** 10.3390/jcm14165746

**Published:** 2025-08-14

**Authors:** Sophia Tsokkou, Alkis Matsas, Ioannis Konstantinidis, Eleni Stamoula, Sofoklis Stavros, Evaggelia Karopoulou, Anastasios Potiris, Theodore Troupis

**Affiliations:** 1 Department of Medicine, Faculty of Health Sciences, Aristotle University of Thessaloniki, 54124 Thessaloniki, Greece; stsokkou@auth.gr (S.T.); ikonsc@auth.gr (I.K.); 2 Laboratory of Experimental Surgery and Surgical Research ‘N.S. Christeas’, Medical School, National and Kapodistrian University of Athens, 11527 Athens, Greece; 3 Department of Clinical Pharmacology, School of Medicine, Aristotle University of Thessaloniki, 54124 Thessaloniki, Greece; 4 Third Department of Obstetrics and Gynecology, Medical School, “Attikon Hospital”, National and Kapodistrian University of Athens, 12462 Athens, Greece; 5 Second Department of Obstetrics and Gynecology, Medical School, “Aretaieion” University Hospital, National and Kapodistrian University of Athens, 11527 Athens, Greece; 6 Department of Anatomy, Faculty of Health Sciences, Medical School, National and Kapodistrian University of Athens, 75 Mikras Asias Str., 11627 Athens, Greece

**Keywords:** ovarian cancer, talcum powder, talcum, genital powder

## Abstract

Ovarian cancer (OC) is a highly lethal gynecologic malignancy, frequently diagnosed at advanced stages due to its silent onset and nonspecific clinical presentation. The use of talc-containing powders in the genital area has long been suspected to contribute to the development of OC through inflammatory and irritative pathways; however, the association remains controversial. This scoping review aims to decode the link between talc powder exposure and ovarian cancer by synthesizing findings from epidemiologic studies, public awareness surveys, and laboratory investigations. Epidemiologic analyses reveal that the use of genital powders is associated with a, modestly, 30–32% increase in the risk of OC, with similar risk patterns observed across racial subgroups. In contrast, studies on uterine cancer yield largely null associations after adjusting for confounders. Awareness surveys consistently report that only about 23% of respondents recognize talc use as a risk factor. Laboratory studies demonstrate that the dominant class of talc particles in commercially available powders—characterized by an aspect ratio of 1–3.9 and an area of 1–400 μm^2^—is nearly identical to those retrieved from pelvic tissues in OC patients, supporting the hypothesis of migration via retrograde and lymphatic pathways. The collective evidence supports the biological plausibility that talc from genital powders can migrate to pelvic tissues and potentially foster oncogenic inflammation. Further methodologically rigorous prospective and mechanistic studies are warranted to clarify the causal relationship and inform targeted public health and regulatory interventions.

## 1. Introduction

Ovarian cancer (OC) is a highly lethal gynecologic malignancy, frequently diagnosed at advanced stages due to its silent onset and nonspecific clinical presentation. The World Ovarian Cancer Coalition states that OC is the 8th most common cancer among the female population worldwide and ranks 8th among death rates. According to Globocan’s 2022 projections, by 2050, the number of women globally diagnosed with OC will surpass 503,448. The number of deaths from OC annually are projected to increase to 350,956, an increase of almost 70% in comparison to 2022 [1].

### 1.1. Survival Rate

According to data from SEER 21 for the period from 2015 to 2021, the 5-year relative survival rate for OC was 51.6%. This indicates that slightly more than half of the individuals diagnosed with ovarian cancer are expected to survive for at least five years following their diagnosis (excluding fatalities from unrelated events). The survival rates of patients are subject to significant variation, due to heterogeneity of patient characteristics. Outcomes are significantly influenced by factors such as age, overall health status, cancer stage at diagnosis, performance status score, and responses to treatment. Although these statistics offer a broad perspective, they cannot be used to anticipate the experiences of individual patients [2]. Only one-fifth of ovarian cancers are diagnosed at an early stage; however, early detection is associated with a five-year survival rate of approximately 94% [3]. The stage at diagnosis is a critical prognostic factor, as it directly reflects the extent of disease dissemination and significantly influences survival outcomes. When cancer is confined to the primary site (localized stage), the five-year relative survival rate is an encouraging 91.7%, and approximately 20% of cases are diagnosed in this category. At a regional stage, characterized by metastasis to regional lymph nodes, in which 19% of cases are diagnosed, the five-year survival rate decreases to 70.7%. In contrast, diagnosis at a distant stage, marked by metastases to other organs, is associated with a markedly reduced five-year survival rate of 31.8%, accounting for approximately 55% of cases. Additionally, for the 6% of cases that remain unstaged, the estimated five-year relative survival rate is 37.0% [2,4].

### 1.2. Symptomatology

Early-stage detection of OC is a challenging task due to the fact that it frequently develops silently and spreads throughout the abdomen before causing evident clinical signs and symptoms. Alterations in dietary patterns, such as early satiety or diminished appetite, may occur alongside symptoms including pelvic or abdominal pain, bloating, or discomfort. Abnormal vaginal discharge or bleeding, particularly in the absence of regular menstrual cycles or following menopause, may also serve as a warning sign. Bowel changes, such as diarrhea or constipation, frequent urination, and an increase in abdominal size are additional prevalent symptoms [5,6]. More specifically, OC symptomatology progresses in severity as the disease advances through its stages. In accordance to FIGO (International Federation of Gynecology and Obstetrics) staging, in stage I, cancer is confined to the ovaries, with symptoms including mild bloating and general discomfort in the abdominal and pelvic region. In stage II, cancer spreads within the pelvis, leading to more noticeable abdominal pain, increased bloating, and frequent urination. In stage III, the disease extends beyond the pelvis into the abdomen. Symptoms can become more severe and include prominent abdominal pain, persistent back pain, significant bloating, early satiety, and fatigue. Stage IV represents the most advanced stage, where cancer spreads to distant organs, often causing severe symptoms such as abdominal swelling, ascites, and other systemic effects. Early identification of these symptoms can be crucial for timely intervention and improved outcomes [7].

### 1.3. Types of Ovarian Cancer

**Figure 1:**
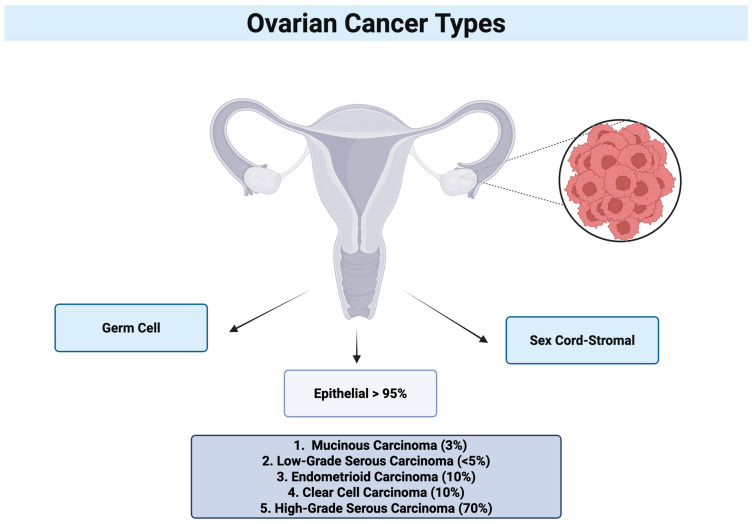
Ovarian cancer [10].

The classification of OC is based on the cells of origin and their behavior. There are three primary cell types of the source of the most prevalent OC types: epithelial, germ, and sex cord–stromal cells (Figure 1). OC encompasses both epithelial and non-epithelial malignancies. Epithelial ovarian cancer represents the predominant histological subtype, accounting for more than 95% of cases. In contrast, non-epithelial ovarian cancers are relatively rare, comprising approximately 5% of cases, and include germ cell tumors, sex cord–stromal tumors, and small cell ovarian carcinomas [8]. Epithelial cells are responsible for the outer membrane of the ovaries, and the majority of OC instances, including epithelial cell tumors, are derived from this layer. Germ cells are responsible for the production of ova and, rarely, they can develop ovarian tumors. Sex cord–stromal cells are responsible for the production of hormones, including estrogen and androgens, and provide support to the ovaries. Although rare, tumors that originate from this region can affect hormone levels [7,8,9].

#### 1.3.1. Epithelial Ovarian Carcinoma

Approximately 80% of women diagnosed with this type of cancer present with advanced-stage disease at the time of diagnosis. Subtypes of epithelial ovarian cancer include serous carcinoma (70–80%), endometrioid carcinoma (10%), clear cell carcinoma (10%), and mucinous carcinoma (3%).

Serous OC, the most prevalent epithelial subtype, includes both high- and low-grade cancers. High-grade serous OC is characterized by aggressive tumor cells believed to originate in the fallopian tube, whereas low-grade serous OC is slower-growing and often resistant to chemotherapy, requiring debulking surgery. Endometrioid OC, associated with endometriosis and Lynch syndrome, accounts for about 10% of epithelial ovarian cancers and is typically diagnosed at earlier stages. Clear cell OC, comprising 5–10% of cases, often affects individuals with endometriosis and is, usually, resistant to chemotherapy. Mucinous OC, the rarest subtype, is usually localized and has a favorable prognosis [9].

Other cancers similar to epithelial include primary peritoneal carcinoma (PPC) and fallopian tube cancer. PPC originates in the peritoneal lining and resembles epithelial OC in presentation, symptoms, and treatment. Fallopian tube cancer is rare and closely related to epithelial ovarian cancer, originating in the tubes connecting the ovary to the uterus. Both PPC and fallopian tube cancer share many clinical similarities with epithelial OC, emphasizing the need for precise diagnosis and tailored treatment strategies [9].

#### 1.3.2. Germ Cell Ovarian Carcinoma

Germ cell tumors represent 15–20% of all ovarian malignancies, with malignant germ cell tumors occurring in 2–6% of cases. These tumors predominantly affect adolescent girls and young women and originate from primordial germ cells of the embryonic gonad [11]. The World Health Organization (WHO) classified malignant germ cell tumors in 1973, categorizing them into dysgerminoma, endodermal sinus tumor (yolk sac tumor), immature teratoma, non-gestational choriocarcinoma, embryonal carcinoma, and mixed germ cell tumors [12].

Malignant mixed germ cell tumors contain two or more malignant germ cell components, representing 8% of all germ cell tumors and exhibiting highly aggressive behavior. The most commonly observed combination is dysgerminoma and endodermal sinus tumor (EST), while the rarest combination includes embryonal carcinoma and immature teratoma. Notably, embryonal carcinoma, although rare, demonstrates the greatest malignant potential [12].

#### 1.3.3. Sex Cord–Stromal Carcinomas

Sex cord–stromal tumors are a diverse group of ovarian neoplasms composed of granulosa cells, theca cells, Sertoli cells, Leydig cells, and fibroblasts, either singly or in various combinations. These tumors exhibit varied clinicopathologic features and biological behaviors, which can pose diagnostic challenges. The WHO classifies sex cord–stromal tumors into several categories, including granulosa–stromal cell tumors, Sertoli–stromal cell tumors, and steroid cell tumors. Granulosa–stromal tumors are further divided into adult and juvenile granulosa cell tumors, as well as thecoma–fibroma subtypes, which may present as luteinized or fibrosarcomatous variants. Sertoli–stromal tumors encompass Sertoli–Leydig cell tumors, ranging from well-differentiated to poorly differentiated forms, some exhibiting retiform patterns or heterologous elements. Sex cord–stromal tumors of mixed or unclassified types include gynandroblastoma and sex cord tumors with annular tubules, while steroid cell tumors can originate from stromal luteoma, Hilus cells, or Leydig cells [13,14].

Sex cord–stromal tumors represent approximately 8% of all ovarian tumors, with immunohistochemistry playing a key role in diagnosis. Markers such as inhibin and calretinin have proven highly valuable, alongside CD56, CD99, A103, cytokeratin, and hormone receptors (ER, PR). CD56 is particularly sensitive and helps differentiate sex cord–stromal tumors from other ovarian neoplasms, especially when inhibin and calretinin staining are inconclusive [13].

In Table 1, a comprehensive classification of ovarian cancer types by the WHO is presented, detailing their respective subtypes, pathological characteristics, and prevalence within the patient population.

### 1.4. Risk Factors of Ovarian Carcinoma

**Figure 2:**
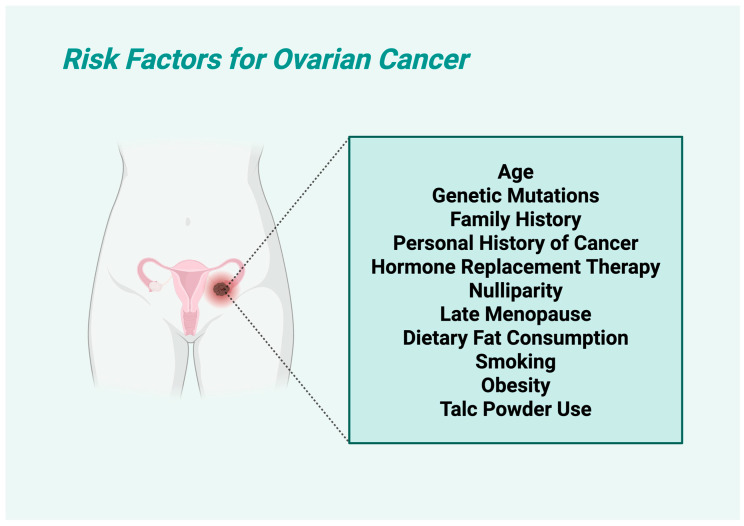
Visualization of risk factors for ovarian cancer.

OC is influenced by multiple factors, with some being well-established and others remaining controversial. Established risk factors include older age (predominantly diagnosed in older women, with approximately ≈75% of diagnoses being in the age group between 60 and 65), with most cases occurring post-menopause; and genetic predisposition, especially mutations in the *BRCA1* and *BRCA2* genes, which significantly increase the risk. The estimated risk of OC for the *BRCA1* mutation is 39 to 46% by age 70 and, for the *BRCA2* mutation, the risk of ovarian cancer is 10 to 27% by the age of 70 [15]. Family history of ovarian, breast, or colorectal cancers is another major key factor, as well as personal history of cancer, which can increase susceptibility [16]. Hormone replacement therapy (HRT), especially prolonged estrogen-only treatments, has been associated with higher risk, as well as nulliparity, which may contribute to increased lifetime ovulatory cycles [17]. More specifically, nulliparous women had a 24% higher risk of OC compared with women who had one child, with a 50% higher risk of endometrioid and a 70% higher risk of clear cell OC [18]. Late menopause is also linked to a higher risk of certain OC subtypes [19]. Dietary fat consumption, particularly from animal sources (especially trans unsaturated fatty acids) [20], has been observed to correlate with increased risk. Smoking plays a dual role, as it significantly raises the likelihood of mucinous OC, but might be protective against other subtypes by lowering estrogen levels [21]. On the other hand, controversial risk factors include obesity, which some studies link to OC, while others find no clear connection. Talc powder use, particularly in the genital area, has long been debated, with mixed findings regarding its carcinogenic potential (Figure 2).

The risk profile of OC is multifactorial, involving both well-established and debated contributors. Table 2 summarizes the principal risk factors associated with ovarian cancer development, categorizing them by their level of scientific consensus and elucidating their respective influences on disease susceptibility. This classification facilitates clearer understanding of epidemiological trends and informs targeted prevention strategies. Additionally Figure 2 shows a visualization of the risk factors of OC.

### 1.5. Mechanistic Pathways of Talc-Induced Ovarian Carcinogenesis

**Figure 3:**
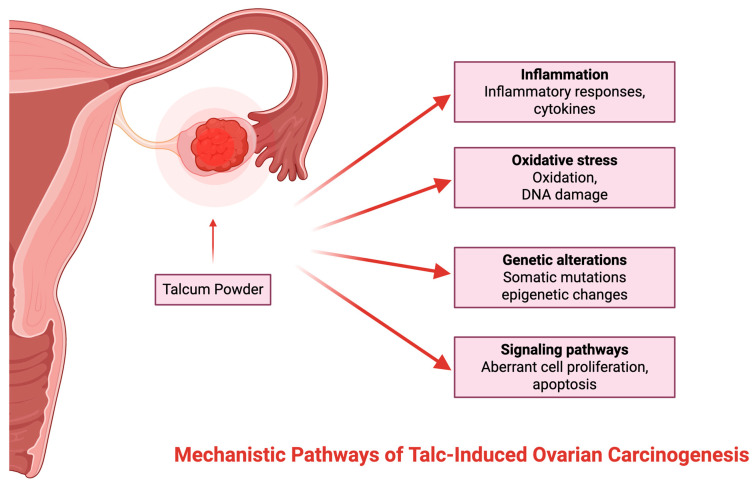
Mechanism of action.

The principal mechanism for talc-induced ovarian cancer is inflammation of the reproductive tract, presumably caused by the migration and embedding of talc particles in ovarian epithelial tissue [22,23,24]. Consequently, elevated oxidative stress levels, DNA damage, and cell division are induced, thereby possibly facilitating carcinogenesis [16]. Research has shown that there have been histopathologic cases of talc identified in the lymph nodes, cervix, uterine corpus, and fallopian tubes via polarized light and scanning electron microscopy [23]. Although the exact mechanism by which talcum powder can contribute to OC development has not yet been fully identified, several theories are circulating [23] (Figure 3).

## 2. Materials and Methods

### 2.1. Search Strategy

A comprehensive literature search was conducted to identify studies examining the association between talcum powder exposure and ovarian cancer, as well as related uterine cancer risk, public awareness, and mechanistic evidence. PubMed (MEDLINE), Scopus, ScienceDirect, Cochrane Library, and Embase were queried from database inception through 30 June 2025. Search terms combined controlled vocabulary and free-text keywords, including “talcum powder”, “genital powder”, “perineal application”, “ovarian cancer”, “uterine cancer”, “talc AND migration”, and “talc AND inflammation”. Boolean operators were used to refine results, and reference lists of retrieved articles were hand-searched to capture additional relevant publications.

### 2.2. Eligibility Criteria

Studies were eligible for inclusion if they met all of the following criteria: (i) published in a peer-reviewed journal in English; (ii) reported original data on human subjects or ex vivo human tissues; and (iii) investigated genital talc use in relation to ovarian or uterine cancer risk, public awareness of talc as an ovarian cancer risk factor, or the morphometric characterization of talc particles in pelvic tissues. Eligible study designs encompassed case–control and cohort epidemiologic investigations, cross-sectional awareness surveys, mechanistic laboratory studies employing microscopy or elemental analysis, and case series or reports with quantitative particle measurements. Excluded were narrative reviews, conference abstracts, animal or in vitro-only studies, editorials, protocols, and publications lacking primary outcome data.

### 2.3. Study Selection and Data Extraction

**Figure 4:**
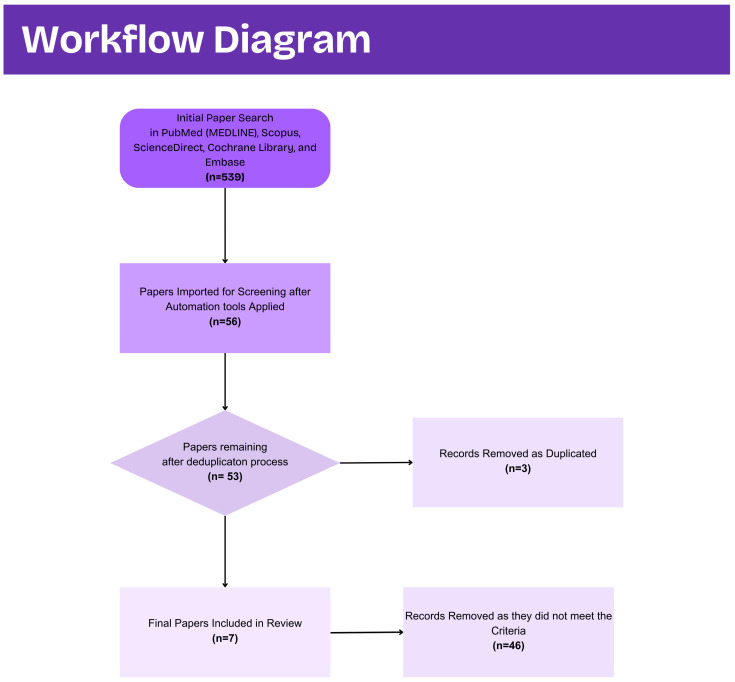
Workflow diagram.

Two investigators (S.T. and I.K.) independently screened titles and abstracts in a double-blinded manner. Full texts were retrieved for all records deemed potentially relevant. Each full-text article was then assessed against the predefined eligibility criteria; disagreements were resolved through discussion with a third reviewer (A.M.). A standardized data extraction template captured the following variables: first author and year of publication, study design, sample size and demographics, talc exposure definition (use, frequency, duration), cancer outcome measures (incidence, histotype), effect estimates (odds ratios, hazard ratios, confidence intervals), and laboratory morphometric parameters (particle aspect ratio, area, elemental composition). Figure 4 captures the screening process performed.

### 2.4. Data Synthesis

Given the heterogeneity of study designs, exposure assessments, and outcome definitions, a narrative synthesis approach was adopted, rather than a quantitative meta-analysis. Findings were grouped into three domains—epidemiologic risk estimates, public awareness surveys, and mechanistic laboratory evidence—and summarized in structured tables. Key methodological features, effect magnitudes, and limitations were mapped to highlight consistencies and discrepancies across studies. This framework facilitated the identification of research gaps and informed recommendations for future prospective and mechanistic investigations.

## 3. Results and Discussion for Talcum Powder

Based on the search performed, it was identified that the risk of OC may be marginally elevated with use of genital powder, which frequently contains talcum powder. This is especially significant for aggressive histologic subtypes. However, the results regarding the danger of uterine cancer continue to be inconsistent.

In women who have ever used genital powders, the risk of ovarian malignancy is 30–32% higher, according to epidemiologic studies. Nevertheless, the risk is reduced when confounding variables such as body mass index and demographic traits are rigorously controlled. In some analyses, frequent and long-term use may indicate a marginally higher risk (e.g., a 12% increased risk in long-term users in one pooled study); however, these associations are not always statistically significant [22].

Talcum powder use is not typically identified as a contributing factor in studies that evaluate public awareness of ovarian cancer risk factors. Thus, educational awareness that is specifically tailored to the intended audience is crucial [23].

Concurrently, laboratory investigations offer compelling evidence of talc’s biophysical migration. Most of the talc particles in commercially available baby powders are small, essentially isodiametric particles with similar aspect ratios and areas. The morphology of these particles is consistent with that of those found in the pelvic tissues of women with ovarian carcinoma, as demonstrated by studies conducted using polarized light microscopy, scanning electron microscopy, and energy-dispersive X-ray analysis [24,25].

### 3.1. Feminine Hygiene Products and Talcum Powder

The study of Llanos AAM et al., 2023, investigates a variety of personal care products (PCPs), with a particular emphasis on feminine hygiene products, which contain talcum powder as a highlighting component. While the research is not exclusively focused on talcum powder, it does acknowledge that certain feminine hygiene products contain talc. In previous research studies, these products attracted attention for their potential associations with adverse health outcomes, as they can be used as douches or for perineal applications. Previous studies have suggested that the use of talcum powder in these contexts might contribute to alterations in the vaginal microbiome, an increased risk of uterine fibroids, and even links to cancers related to the reproductive system [25].

Additionally, the paper unveils major variations in usage patterns. More precisely, it emphasizes that non-Hispanic Black (NHB), Hispanic, and multiracial or other women tend to report substantially higher usage of feminine hygiene products, including those containing talcum powder, than non-Hispanic White (NHW) women. This differential utilization raises concerns about unequal chemical exposures, as a higher frequency of use may result in a greater cumulative burden of potential endocrine-disrupting chemicals or contaminants that may be present in talc-based products [25].

Moreover, understanding these usage patterns is critical within the broader context of environmental health. There are documented concerns regarding talcum powder, primarily due to the possibility of contamination with asbestos if it is extracted and processed without sufficient quality control. While modern refining techniques aim to ensure asbestos-free talc, the legacy of such contamination concerns continues to fuel both consumer apprehension and legal challenges. The significance of examining cumulative chemical exposures, particularly among populations that are already at a higher risk of adverse health outcomes, is emphasized in the study through the use of talcum powder as an example [25].

### 3.2. Body Powders and Ethnicity

Moreover, the study of Davis C.P. et al. 2025 investigates the use of body powders applied to the genital area (including talcum powder) in relation to higher risk of OC development. Data were pooled from five studies within the OCWAA consortium, encompassing 620 African American cases with 1146 controls, and 2800 White cases with 6735 corresponding controls. In this research, genital powder use was defined broadly to include all types of powders (such as baby, talcum, deodorizing, or cornstarch powders) that were applied directly to the genital, perineal, or rectal areas, or indirectly through sanitary products [26].

The overall prevalence of ever using genital powder was slightly higher among African American women compared with White women. Among ovarian cancer cases, 35.8% of African American women reported having used genital powder compared with 29.5% of White women. A similar pattern was seen in the control groups. When evaluating the association with cancer risk, the study found that the use of genital powder was linked to an approximately 32% increase in the odds of developing OC overall. When the data were analyzed by race, White women showed a statistically significant 36% increase in risk (odds ratio = 1.36), while African American women demonstrated a 22% increase (odds ratio = 1.22), though the difference between the groups was not statistically significant [26].

The study also assessed associations by tumor histotype. Both African American and White women who reported using genital powder had higher risks for high-grade serous OC, with African American women exhibiting an odds ratio of 1.31, and White women an odds ratio of 1.33. In contrast, for non-high-grade serous histotype, a significant association was observed in White women (odds ratio = 1.38), but not in African American women (odds ratio = 1.05), suggesting that the relationship between powder use and OC risk might differ by tumor subtype, especially among African American women [26].

When the researchers examined patterns of use, including frequency of product application and duration of use, no clear dose–response relationship was noted. Whether women used genital powders less than or more than once per week, the relative increase in risk was similar. Similarly, when duration was compared between those who used the powder for 20 years or less versus more than 20 years, the risk estimates did not show a consistent trend. Additionally, the population attributable risk (PAR) calculations, indicating the proportion of OC cases that could be linked to genital powder use, were comparable between African American women (approximately 7.5%) and White women (approximately 6.2%) [26].

The study suggests that the practice of using genital powders (including those containing talcum powder) is associated with an increased risk of epithelial ovarian cancer in both African American and White women. Although genital powder use is more commonly reported among African American women, the magnitude of the associated risk appears similar across the two groups. The findings also indicate that the increased risk is not driven by a dose–response effect in terms of frequency or duration, pointing to the need for further research to refine exposure assessments and understand additional factors that may influence risk [26].

Additionally, the study of Radu C.A. et al. 2023 quantitatively assessed ovarian cancer risk factor awareness using both recall and recognition formats in a young, ethnically diverse British population. Among the risk factors evaluated, genital use of talcum powder was notably underrecognized. Specifically, only approximately 0.5% of participants mentioned talcum powder in the genital area when prompted in a free-text recall task. In contrast, even when provided with a list of potential risk factors, only 23.0% of respondents correctly recognized talcum powder use as being associated with an increased risk of ovarian cancer. This low level of both unprompted recall and prompted recognition indicates that awareness of the association between genital talc exposure and ovarian carcinogenesis is minimal among the public surveyed [27].

These findings are particularly relevant in the context of the ongoing scientific debate. A possible function for talc in initiating inflammatory or other carcinogenic processes within the ovarian epithelium has been suggested by the consistent reports of associations between genital talc use and an elevated risk of epithelial ovarian cancer in recent retrospective case–control studies. Nevertheless, this association has not been consistently observed in prospective cohort studies, which are less susceptible to recall bias. The current controversy in the literature regarding the etiologic function of talc may be attributed to the discrepancy between retrospective and prospective data. As a result, the low public awareness of genital talc use as a risk factor for OC seems to be a reflection of this scientific uncertainty [27].

In summary, the study’s data reveal that talcum powder, despite its frequent inclusion in feminine hygiene products and prior epidemiologic associations with OC, remains one of the least recognized risk factors among a young and diverse British population, suggesting that further research is necessary to clarify the biological and epidemiological links between talcum powder exposure and OC, and enhanced, evidence-based educational efforts may be crucial to bridge the gap between scientific findings and public understanding [27].

### 3.3. Screening and Awareness of Ovarian Cancer

Additionally, the study of Maryam B et al. 2022, which is a cross-sectional study, was conducted in the western regions of Iran during the years 2020–2021. The study aimed to assess women’s awareness of OC warning signs and risk factors using a modified version of the Ovarian Cancer Awareness Measure (OCAM). A total of 1081 women between 18 and 70 years of age participated by completing an online questionnaire distributed from midwives. The instrument gathered demographic data and evaluated knowledge across several dimensions, including warning signs, risk factors, and awareness of national screening programs [28].

The findings revealed that, while a majority (60.9%) of participants exhibited moderate awareness of OC, key warning signs and risk factors were poorly recognized by many respondents. For instance, symptoms such as persistent bloating, a continuous sense of abdominal fullness, and frequent urination were among the least identified, and the association between the application of talcum powder to the genital area and an increased risk for OC was particularly underrecognized. In contrast, risk factors like a history of ovarian cysts and a family history of ovarian cancer were more widely known. Higher levels of education and a personal or family history of cancer were significantly associated with better awareness. These results underscore a critical need for targeted educational interventions aimed at improving early recognition and prompt medical consultation to potentially enhance outcomes in OC [28].

### 3.4. Epidemiological Insights

Furthermore, the study of O’Brien K.M. et. al. 2021, which is a pooled analysis of four large prospective cohorts (including the Nurses’ Health Study (NHS), Nurses’ Health Study II (NHSII), Sister Study (SIS), and the Women’s Health Initiative Observational Study (WHI-OS)), investigated the association between genital powder use and the risk of invasive uterine cancer. The combined analytic sample comprised 209,185 women with a mean follow-up duration of 14.5 years, during which 3272 incident uterine cancer cases were documented. Approximately 37% of the participants reported the use of genital powder, with distinct variations in prevalence among cohorts (41% in NHS, 52% in WHI-OS, and 26% in both NHSII and SIS). After comprehensive adjustment for confounding variables (body mass index (modeled using a restricted cubic spline), race/ethnicity, education, parity, smoking status, oral contraceptive use, menopausal status, hormone therapy, and other relevant covariates) the multivariable hazard ratio for use versus no use was 1.01 (95% CI: 0.94–1.09). Analyses stratified by frequency (defined as at least once per week) and duration (long-term use defined as more than 20 years) of genital powder use produced similar results, with frequent use yielding an adjusted HR of approximately 1.05 and long-term use demonstrating a borderline increased risk (HR = 1.12, 95% CI: 0.96–1.31) that did not achieve statistical significance [29].

With respect to talcum, the study emphasizes that genital powders often contain talc, a substance that has been hypothesized to promote carcinogenesis through chronic irritation and inflammation of exposed tissues. Although prior research has reported a potential association between talc exposure and ovarian cancer risk, the current analysis does not support a significant link between genital powder (and by extension, talcum) use and uterine cancer risk. Notably, any indication of a positive association was most pronounced during the initial 10 years of follow-up, which might suggest that recent exposure is of greater relevance. However, this temporal signal diminished over longer follow-up intervals, possibly due to exposure misclassification or changes in product formulations (e.g., removal of asbestos contaminants from talc-based products after 1976) [29].

### 3.5. Morphometric Analysis of Talc in Ovarian Carcinoma

The study of Johnson K.E. et. al. 2020 performed a detailed morphometric analysis of talc particles in both commercially available talcum powder (TCBP) and pelvic tissues resected from OC patients using polarized light microscopy (PLM) and scanning electron microscopy (SEM) with energy dispersive X-ray analysis (SEM/EDX). In the TCBP samples, analysis of 400 randomly selected particles revealed that 77.7% had an aspect ratio (AR) between 1 and 3.9, with an area ranging from 1 to 400 µm^2^; 14.0% had an AR between 1 and 2, with an area exceeding 400 µm^2^; and 8.3% had a higher AR (4.0 to >10), with an area between 1 and 200 µm^2^. In comparison, among 200 talc particles analyzed from ovarian carcinoma resected tissues, 83.5% fell within the AR 1–3.9 and area 1–400 µm^2^ category, whereas none were observed in the AR 1–2 with area >400 µm^2^ class, and 16.3% exhibited an AR in the 4–10 range with an area of 1–200 µm^2^. These proportions indicate a striking similarity in the dominant particle morphology between TCBP and the talc found in patient tissues [30].

The resected tissues provided additional statistical information, indicating that the talc particles had a maximum length of 28.9 µm and a size range of 0.9–28.9 µm. The average length was 5.78 µm (SD = 4.02 µm), and the average aspect ratio was 2.49 (SD = 1.51). The average particle length in a subset of cervical tissue from a serous carcinoma case was 6.30 µm (SD = 5.02 µm), with an average width of 2.33 µm (SD = 1.57 µm), an average area of 20.10 µm^2^ (SD = 33.09 µm^2^), and an average aspect ratio of 2.98 (SD = 1.75). These statistical parameters serve to confirm that the preponderance of talc particles in ovarian carcinoma and TCBP tissues are small, roughly isodiametric particles. The hypothesis that talc from these cosmetic products can migrate from the perineum to the upper genital tract is robustly supported by the close relationship in particle size and shape between the talc particles lodged in pelvic tissues and the talc that is externally applied. Talc’s potential to promote carcinogenic processes in ovarian tissues is further emphasized by its propensity to induce chronic inflammation and this migration [30].

Similar to the previous study, the investigation conducted by McDonald S.A. et al. 2019 aimed to evaluate whether talc particles resulting from perineal cosmetic use can migrate to various pelvic organ sites in women diagnosed with OC. Thus, five patient cases with documented perineal talc exposure were analyzed using both polarized light microscopy (PLM) and in situ scanning electron microscopy (SEM) with energy-dispersive X-ray analysis (SEM/EDX) on resected pelvic tissues. The investigators found abundant birefringent talc particles—characterized by typical magnesium and silicon peaks and Mg/Si ratios within 5% of the theoretical value—in multiple pelvic locations, such as the cervix, uterine corpus, fallopian tubes, ovaries, and particularly pelvic lymph nodes. Notably, most of these particles were small, isodiametric, and ranged between 1 and 10 µm in maximum dimension, closely resembling the dominant particle morphology identified in commercially available talcum powder [31].

Quantitative analyses revealed that tissue blocks from talc-exposed patients frequently contained tens to several hundred talc particles per block, with a strong correlation between light microscopy counts and SEM/EDX findings. In contrast, control tissues from patients with no history of talc exposure yielded either minimal or no talc particles, thereby reinforcing the exposure-specific migration pattern. Talc was predominantly localized within macrophages and in fibrovascular stroma, suggesting that its presence may trigger a chronic inflammatory response through lymphatic and retrograde pathways. These findings substantiate the biological plausibility of epidemiologically observed associations between perineal talc use and ovarian carcinogenesis, and highlight the importance of vigilant histopathologic evaluation of pelvic tissues in exposed patients [31].

Table 3 provides a summarized synthesis of current research exploring the relationship between talcum powder exposure and OC, integrating the findings discussed above from epidemiological studies, public awareness surveys, histopathologic analyses, and cohort-based risk assessments.

## 4. Future Directions

To advance our understanding of the potential causal link between genital talcum powder use and ovarian cancer, future research must prioritize large-scale prospective cohort studies that integrate detailed, quantitative exposure assessment with objective biological measurements. By systematically recording product type, frequency of application, duration of use, and documented talc composition over time, these studies can reduce reliance on retrospective recall and improve dose–response modeling. Incorporating serial biospecimen collection, such as peritoneal washings, urine, or blood, will enable validation of self-reported exposure and the development of biomarkers for cumulative talc burden.

Parallel efforts to harmonize histopathologic and particle-mapping methodologies will be essential for comparing findings across laboratories and studies. Establishing consensus guidelines for polarized light microscopy, scanning electron microscopy with energy-dispersive X-ray analysis, and morphometric classification of talc particles in resected ovarian and pelvic tissues will facilitate pooled analyses. Uniform tissue-processing protocols and reporting templates can help quantify tissue burdens accurately, fostering meta-analyses that clarify the relationship between talc morphology and carcinogenic potential.

Mechanistic investigations should focus on how talc particles interact with ovarian epithelial and stromal cells to initiate and sustain chronic inflammation, oxidative stress, and genotoxic damage. Advanced three-dimensional organoid models that recapitulate the ovarian microenvironment will allow researchers to explore dose thresholds for inflammatory activation, characterize downstream signaling pathways, and identify candidate molecular targets for chemoprevention. Parallel ex vivo studies using human fallopian tube and ovarian tissue explants can further validate in vitro findings under conditions that mimic in vivo exposures.

Understanding individual susceptibility will require integrating molecular epidemiology with genetic profiling. Large case-control or cohort studies that couple talc exposure data with germline sequencing can uncover gene-environment interactions in critical pathways such as DNA repair, cytokine signaling, and detoxification. Identifying polymorphisms or epigenetic markers that modify talc-related risk will support personalized risk stratification and targeted surveillance protocols for high-risk subpopulations.

Finally, bridging the gap between scientific evidence and public perception demands rigorously evaluated educational interventions tailored to demographic groups most likely to use talc-containing powders. Randomized trials of culturally sensitive messaging, delivered through community health networks, digital platforms, and healthcare providers, can assess changes in awareness, product choices, and early symptom recognition. Alongside these efforts, collaborative research with industry and regulatory agencies should examine talc raw material sourcing, contamination controls, and alternative formulations, ensuring that consumer safety standards evolve in step with emerging mechanistic and epidemiologic insights.

## 5. Conclusions

The reviewed evidence collectively supports the biologic plausibility that talc, a component of widely used genital powders, can migrate from the perineum to the pelvic organs via retrograde and lymphatic pathways. Epidemiologic studies indicate that the use of genital powder is associated with a slight increase in the risk of ovarian cancer and, to a lesser extent, a borderline association with the risk of uterine cancer in long-term consumers. Social recognition of talcum powder as a risk factor for OC is consistently low, as evidenced by awareness studies that were investigated in the current review, suggesting significant discrepancy between scientific discoveries and public awareness.

The morphology of talc particles in commercial products is analogous to that of pelvic tissues, as confirmed by laboratory analyses that employ advanced microscopy techniques. This evidence is compelling and supports the migration hypothesis. Although these discoveries make a substantial contribution to our comprehension of the potential mechanisms that underlie talc-induced inflammation and carcinogenesis, the current epidemiologic evidence is inconsistent. Consequently, to elucidate the causal relationship and to inform educational and regulatory interventions that are designed to reduce exposure, it is necessary to conduct additional research, including methodologically rigorous prospective studies and refined laboratory investigations.

## Figures and Tables

**Table 1:** Taxonomic overview of ovarian cancer classifications by the World Health Organization [9].

Type	Subtypes	Characteristics	Prevalence
Epithelial Ovarian Carcinoma	Serous (high-grade and low-grade) Endometrioid Clear Cell, Mucinous PPC Fallopian Tube Cancer	- Originates from outer ovarian membrane - High-grade serous: aggressive, fallopian tube origin - Low-grade serous: slow-growing, chemo-resistant - Endometrioid: linked to endometriosis, Lynch syndrome - Clear Cell: chemo-resistant - Mucinous: localized, favorable prognosis - PPC and Fallopian Tube: similar presentation and treatment	~95% of all OC cases
Germ Cell Ovarian Carcinoma	Dysgerminoma Yolk Sac Tumor Immature Teratoma Choriocarcinoma Embryonal Carcinoma Mixed Germ Cell Tumors	- Originates from ova-producing cells - Affects adolescents and young women - Mixed tumors are aggressive - Embryonal carcinoma has highest malignant potential	2–6% of OC cases
Sex Cord–Stromal Carcinoma	Granulosa–Stromal (adult/juvenile) Thecoma–Fibroma Sertoli–Leydig Gynandroblastoma Steroid Cell Tumors	- Originates from hormone-producing/support cells - Diverse histology and behavior - Diagnosed using immunohistochemistry (e.g., inhibin, calretinin, CD56) - May affect hormone levels	~8% of ovarian tumors

**Table 2:** Summary of established and controversial risk factors for ovarian cancer.

Risk Factor	Description	Impact on OC Risk
Age	Most diagnoses occur post-menopause; ~75% between ages 60–65 [15]	Strongly increases risk [15]
Genetic Mutations	BRCA1 (39–46% risk by age 70), BRCA2 (10–27% risk by age 70) [15]	Significantly increases lifetime risk [15]
Family History	History of ovarian, breast, or colorectal cancers [16]	Increases susceptibility [16]
Personal History of Cancer	Prior malignancies [16]	Elevates risk [16]
Hormone Replacement Therapy	Prolonged estrogen-only treatment [17]	Associated with increased risk [17]
Nulliparity	No childbirth; increased ovulatory cycles [17,18]	24% higher overall risk; 50% higher for endometrioid, 70% for clear cell OC [17,18]
Late Menopause	Extended exposure to estrogen [19]	Associated with higher risk of certain subtypes [19]
Dietary Fat Consumption	High intake of animal fats, especially trans fats [20]	Correlated with increased risk [20]
Smoking	Raises risk of mucinous OC; may lower estrogen levels [21]	Dual effect: increases mucinous OC risk, possibly protective for other subtypes [21]
Obesity	Excess body weight [20]	Mixed findings; some studies show increased risk, others do not [20]
Talc Powder Use	Genital application [22,23,24,25,26,27,28]	Debated carcinogenic potential; inconsistent findings [22,23,24,25,26,27,28]

**Table 3:** Talcum powder use and its association with ovarian and uterine cancer risk and awareness.

Author, Year	Key Findings	Population Studied	Impact on OC Risk
Llanos AAM et al. (2023) [25]	Talc in feminine hygiene products may affect vaginal microbiome and reproductive health	NHB, Hispanic, multiracial, and NHW women	Higher usage among NHB and Hispanic women; potential for unequal chemical exposure
Davis C.P. et al. (2021) [26]	Genital powder use associated with a 32% higher risk overall; stronger effect in White women (OR = 1.36) than NHB women (OR = 1.22)	620 NHB cases, 2800 White cases from OCWAA consortium	Elevated risk for high-grade serous OC; no clear dose–response pattern
Radu C.A. et al. (2023) [27]	Talcum powder use is poorly recognized as a risk factor in public awareness surveys	Young, ethnically diverse British population	Very low awareness: 0.5% recall, 23% recognition
Maryam B et al. (2022) [28]	Moderate overall awareness of OC; talcum powder risk poorly recognized; education and cancer history linked to better awareness	1081 women aged 18–70 in western Iran	Highlights need for targeted education to improve recognition of OC symptoms and risks
O’Brien K.M. et al. (2021) [29]	No significant association between genital powder use and uterine cancer risk; borderline increase in long-term users not statistically significant	209,185 women from four prospective cohorts (NHS, NHSII, SIS, WHI-OS)	Suggests limited relevance of talc exposure for uterine cancer; possible early exposure signal
Johnson K.E. et al. (2020) [30]	Talc particles in ovarian carcinoma tissues closely match those in commercial talcum powder in size and shape	200 talc particles from OC tissues vs. 400 from TCBP samples	Supports migration hypothesis; reinforces biological plausibility of talc-induced inflammation
McDonald S.A. et al. (2019) [31]	Talc particles found in multiple pelvic organs of exposed OC patients; absent in controls	5 OC patients with documented perineal talc exposure	Confirms tissue migration and chronic inflammatory response; strengthens causal inference

## Data Availability

No new data were created or analyzed in this study.

## References

[1] World Ovarian Cancer Coalition Ovarian Cancer Key Stats. https://worldovariancancercoalition.org/about-ovarian-cancer/key-stats/#:~:text=According%20to%20Globocan’s%202022%20projections,of%20almost%2070%25%20from%202022.

[2] (2025). Cancer Stat Facts: Ovarian Cancer.

[3] Trinidad C.V., Tetlow A.L., Bantis L.E., Godwin A.K. (2020). Reducing Ovarian Cancer Mortality Through Early Detection: Approaches Using Circulating Biomarkers. Cancer Prev. Res..

[4] American Cancer Society (2022). Ovarian Cancer Early Detection, Diagnosis, and Staging.

[5] Bankhead C., Collins C., Stokes-Lampard H., Rose P., Wilson S., Clements A., Mant D., Kehoe S.T., Austoker J. (2008). Identifying symptoms of ovarian cancer: A qualitative and quantitative study. BJOG.

[6] Rossing M.A., Wicklund K.G., Cushing-Haugen K.L., Weiss N.S. (2010). Predictive Value of Symptoms for Early Detection of Ovarian Cancer. JNCI J. Natl. Cancer Inst..

[7] Stewart C., Ralyea C., Lockwood S. (2019). Ovarian Cancer: An Integrated Review. Semin. Oncol. Nurs..

[8] Arora T., Mullangi S., Vadakekut E.S., Lekkala M.R. (2024). Epithelial Ovarian Cancer.

[9] Kaku T., Ogawa S., Kawano Y., Ohishi Y., Kobayashi H., Hirakawa T., Nakano H. (2003). Histological classification of ovarian cancer. Med. Electron Microsc..

[10] Tsokkou S. (2025). Created in BioRender. Ovarian Cancer Types. https://BioRender.com/undefined.

[11] Pectasides D., Pectasides E., Kassanos D. (2008). Germ cell tumors of the ovary. Cancer Treat. Rev..

[12] Goyal L.D., Kaur B., Kumari Badyal R. (2019). Malignant Mixed Germ Cell Tumors of the Ovary: A Series of Rare Cases. J. Reprod. Infertil..

[13] Esheba G.E. Sex Cord Stromal Ovary Tumor Pathology Sex Cord-Stromal Tumors Definition. https://emedicine.medscape.com/article/1627984-overview.

[14] Horta M., Cunha T.M. (2015). Sex cord-stromal tumors of the ovary: A comprehensive review and update for radiologists. Diagn. Interv. Radiol..

[15] Ramus S.J., Gayther S.A. (2009). The Contribution of *BRCA1* and *BRCA2* to Ovarian Cancer. Mol. Oncol..

[16] Menon U., Karpinskyj C., Gentry-Maharaj A. (2018). Ovarian Cancer Prevention and Screening. Obstet. Gynecol..

[17] (2024). Does Hormone Replacement Therapy (HRT) Increase the Risk of Cancer?.

[18] Burns E.M. (2018). Nulliparity linked to higher risk of clear cell & endometrioid ovarian tumors. Focus on Ovarian Cancer.

[19] Shen F., Chen S., Gao Y., Dai X., Chen Q. (2017). The prevalence of malignant and borderline ovarian cancer in pre-and post-menopausal Chinese women. Oncotarget.

[20] Gilsing A.M.J., Weijenberg M.P., Goldbohm R.A., Van Den Brandt P.A., Schouten L.J. (2011). Consumption of dietary fat and meat and risk of ovarian cancer in the Netherlands Cohort Study. Am. J. Clin. Nutr..

[21] Baron J.A., Nichols H.B., Anderson C., Safe S. (2021). Cigarette smoking and estrogen-related cancer. Cancer Epidemiol. Biomark. Prev..

[22] Crawford L., Reeves K.W., Luisi N., Balasubramanian R., Sturgeon S.R. (2012). Perineal powder use and risk of endometrial cancer in postmenopausal women. Cancer Causes Control.

[23] Micha J.P., Rettenmaier M.A., Bohart R., Goldstein B.H. (2022). Talc powder and ovarian cancer: What is the evidence?. Arch. Gynecol. Obstet..

[24] Wentzensen N., O’Brien K.M. (2021). Talc, body powder, and ovarian cancer: A summary of the epidemiologic evidence. Gynecol. Oncol..

[25] Llanos A.A.M., Rockson A., Getz K., Greenberg P., Portillo E., McDonald J.A., Teteh D.K., Villasenor J., Lozada C., Franklin J. (2023). Assessment of personal care product use and perceptions of use in a sample of US adults affiliated with a university in the Northeast. Environ. Res..

[26] Davis C.P., Bandera E.V., Bethea T.N., Camacho F., Joslin C.E., Wu A.H., Beeghly-Fadiel A., Moorman P.G., Myers E.R., Ochs-Balcom H.M. (2021). Genital powder use and risk of epithelial ovarian cancer in the ovarian cancer in women of African Ancestry consortium. Cancer Epidemiol. Biomark. Prev..

[27] Radu C.A., Matos de Melo Fernandes N., Khalfe S., Stordal B. (2023). Awareness of ovarian cancer symptoms and risk factors in a young ethnically diverse British population. Cancer Med..

[28] Maryam B., Fatemeh S., Nourossadat K., Saeideh N., Giti O. (2022). Women’s awareness of ovarian cancer risk factors and symptoms in Western Iran in 2020–2021. BMC Womens Health.

[29] O’Brien K.M., Tworoger S.S., Harris H.R., Trabert B., Weinberg C.R., Fortner R.T., D’Aloisio A.A., Kaunitz A.M., Wentzensen N., Sandler D.P. (2021). Genital powder use and risk of uterine cancer: A pooled analysis of prospective studies. Int. J. Cancer.

[30] Johnson K.E., Popratiloff A., Fan Y., McDonald S., Godleski J.J. (2020). Analytic comparison of talc in commercially available baby powder and in pelvic tissues resected from ovarian carcinoma patients. Gynecol. Oncol..

[31] Mcdonald S.A., Fan Y., Welch W.R., Cramer D.W., Godleski J.J. (2019). Migration of Talc From the Perineum to Multiple Pelvic Organ Sites. Am. J. Clin. Pathol..

